# Estimation of systolic blood pressure by Random Forest using heart sounds and a ballistocardiogram

**DOI:** 10.1038/s41598-022-22205-0

**Published:** 2022-10-13

**Authors:** Rafael Gonzalez-Landaeta, Brenda Ramirez, Jose Mejia

**Affiliations:** Department of Electrical and Computer Engineering, Autonomous University of Ciudad Juarez, 32310 Ciudad Juárez, Mexico

**Keywords:** Machine learning, Data processing

## Abstract

Cuffless blood pressure measurement enables unobtrusive and continuous monitoring that can be integrated with wearable devices to extend healthcare to non-hospital settings. Most of the current research has focused on the estimation of blood pressure based on pulse transit time or pulse arrival time using ECG or peripheral cardiac pulse signals as proximal time references. This study proposed the use of a phonocardiogram (PCG) and ballistocardiogram (BCG), two signals detected noninvasively, to estimate systolic blood pressure (SBP). For this, the PCG and the BCG were simultaneously measured in 21 volunteers in the rest, activity, and post-activity conditions. Different features were considered based on the relationships between these signals. The intervals between S1 and S2 of the PCG and the I, J, and K waves of the BCG were statistically analyzed. The IJ and JK slopes were also estimated as additional features to train the machine-learning algorithm. The intervals S1-J, S1-K, S1-I, J-S2, and I-S2 were negatively correlated with changes in SBP (p-val < 0.01). The features were used as explanatory variables for a regressor based on the Random Forest. It was possible to estimate the systolic blood pressure with a mean error of 3.3 mmHg with a standard deviation of ± 5 mmHg. Therefore, we foresee that this proposal has potential applications for wearable devices that use low-cost embedded systems.

## Introduction

Arterial blood pressure (ABP) is a reliable indicator of health conditions. Current gold standard noninvasive methods rely on the use of inflatable cuff-based systems, which may be uncomfortable for some subjects. To tackle this, different cuff-less approaches have been proposed, mainly based on the estimation of pulse transit time (PTT), pulse arrival time (PAT), and pulse wave velocity (PWV). This implies multimodal measurement approaches^1^, the most common being those where the ECG is used as a proximal timing reference signal versus the peripheral pulse. In these cases, the time between the R-wave and different points of the pulse signal (peak, foot, and slope) was measured to determine the correlation between ABP and PTT^2,3^. This method is termed PAT and includes the pre-ejection period (PEP) (PAT = PTT + PEP)^4^, which depends on the isovolumetric contraction in the left ventricle^5^ and is influenced by sympathetic activity^6^. PEP changes independently of ABP^2,7^, and it is not easy to estimate it.

Other biosignals have been considered proximal time references; the most common are photoplethysmography (PPG)^8,9^, phonocardiography (PCG)^10^, ballistocardiography (BCG)^7,11^, and seismocardiography (SCG)^12^. With these signals, the PEP is excluded, and the PTT is estimated by measuring the time between some waves of these signals and the peak, foot, or slope of the peripheral pulse signal.

The PCG is a good indicator of the onset of mechanical systole^13,14^, and the analysis of its main waves provides information about blood pressure (systolic, diastolic, and mean)^15^. Regarding the use of PCG as a proximal timing reference signal, the PTT is usually estimated by using the PPG as a distal timing signal, and it has been demonstrated that the correlation with ABP is comparable when using the ECG as reference^10^. However, the morphology of the PPG signal can be modified by the effects of aging^16^ and by the peripheral arterial stiffness^17^, bringing about misleading results when using PTT as an ABP indicator.

**Figure 1:**
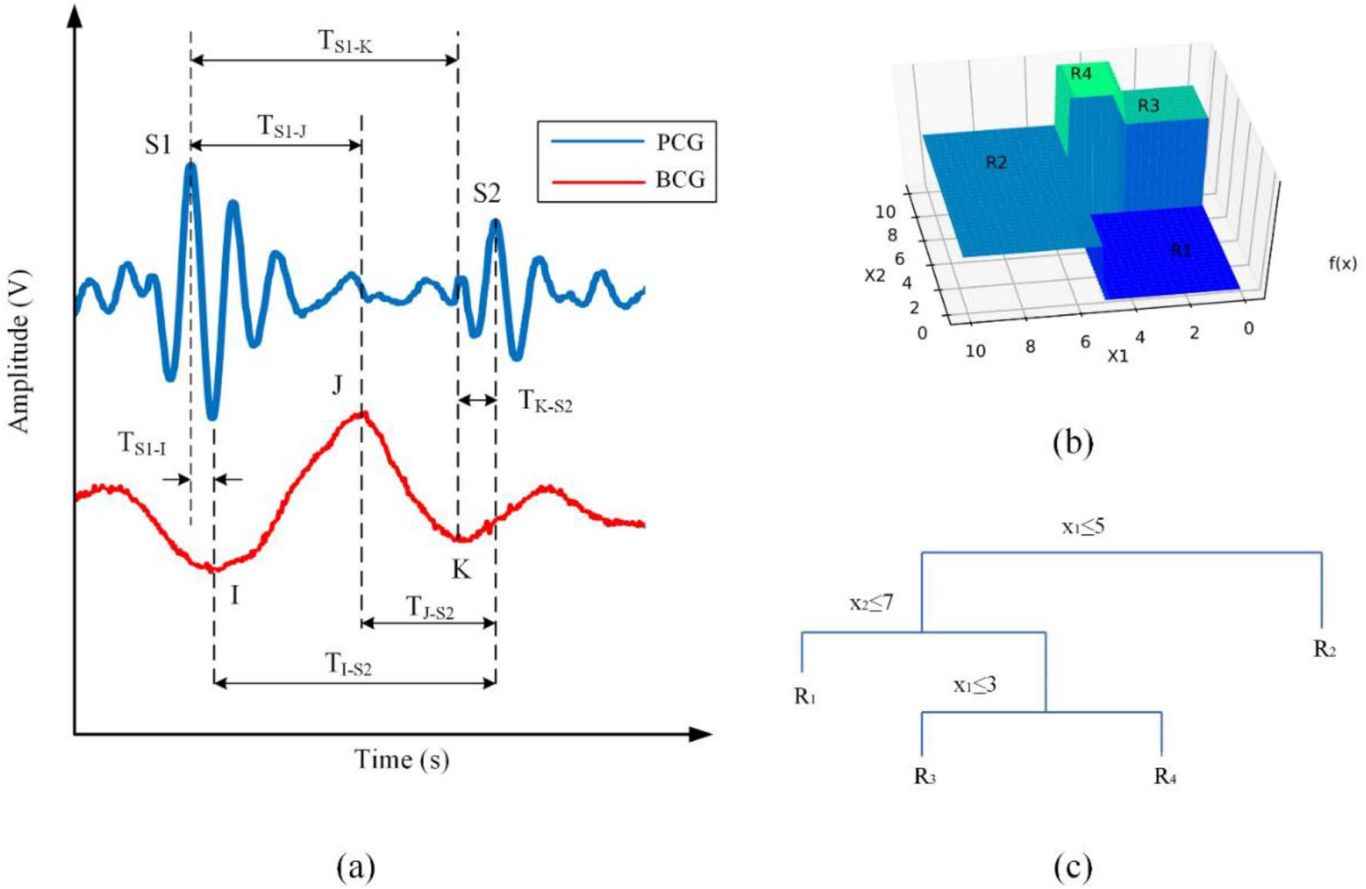
(**a**) Time intervals between the PCG and the BCG. (**b**) Example of a tree model, where the tree input space is a box of ${\mathbb{R}}^{2}$ and a partition of the input space, and (**c**) the associated decision tree.

A different approach is proposed in this paper, where it is not necessary to detect the peripheral pulse. The change in systolic ABP is estimated by detecting the time delay between the two main sounds (S1, S2) of the PCG and the main waves (I, J, K) of the BCG. This idea stems from the fact that changes in blood pressure have little effect on the onset of S1 and S2. However, they do have an important influence on the genesis of the main waves of the BCG. In the case of PCG, S1 is due to the simultaneous closure of the atrioventricular valves, so its correlation with blood pressure is not as evident compared with that of S2, whose spectrum has been shown to have a significant correlation with systolic blood pressure (SBP)^15,18^. In the case of BCG, pressure gradients in the ascending and descending aorta define both the amplitudes and the onsets of I, K, and K waves^19^. In this sense, we believe that a change in systolic pressure causes a variation in the time delay between the main waves of the PCG and the BCG. To validate this, both signals are detected simultaneously, and the time relationship between the main waves of PCG and BCG is analyzed. To conduct the measurements in a simple way, in this work, the PCG is measured at the sternum, while the BCG is measured using a weighing scale. However, the intention is that these signals can be detected using wearable systems capable of detecting BCG at the sternum, using methods such as that proposed by Wiens et al.^20^, so both signals (PCG and BCG) could be measured at the same point. From the PCG, both S1 and S2 are used as proximal time references, and the time delay with respect to each I, J, and K wave of the BCG is estimated; see Fig. 1a. In addition, these time intervals are used as explanatory variables in a Random Forest regressor (RF) to estimate systolic blood pressure. The RF model provides more information because it evaluates the importance of each explanatory variable over the response variable (i.e., systolic blood pressure). To the best of our knowledge, these two signals have not been used together to assess the correlation with the ABP, so we consider that the results derived from this research may be relevant for future work related to the estimation of ABP using cuff-less methods.

This manuscript is organized as follows: Sect. “Random forest theory” provides a brief introduction to the RF model. Section “Methods” describes the setup used for signal acquisition, signal processing, and measurement protocol. Section “Results and discussion” shows the experimental results and discussion, and Sect. “Conclusion” draws the main conclusions.

## Random forest theory

### Regression trees

Classification and regression tree models work by recursively partitioning their input space into M decision regions^21^. They are named such in part because such a partition is generally represented by a tree, with its leaf denoting different regions. Let $f\left({\varvec{x}}\right)$ be the output of the tree to an input vector ${\varvec{x}}$. Then the model can be written as:

1 $$f\left({\varvec{x}}\right)=\sum_{m=1}^{M}{w}_{m}I({\varvec{x}}\in {R}_{m})$$

where $I(\cdot )$ is an indicator function, ${R}_{m}$ is the *m*th region, and ${w}_{m}$ is its associated weight. Figure 1b shows an example of an input space partitioned into four regions by the tree of Fig. 1c. Although tree models are well suited to large data sets and they handle outliers relatively well, they suffer from a lack of accuracy compared to other models. In addition, trees are known to be unstable due to small changes to the input data, and they are considered high variance estimators.

### Random forest

Random Forests are procedures that use an ensemble of decision trees and are recognized for their high prediction performance when dealing with real problems^22^. In these models, each tree was constructed using a randomly chosen subset of the training set through a random vector $\Theta$. An RF model tries to reduce the variance of the regression tree model by averaging many tree estimates. Considering that the model uses T decision trees, $\{ {f}_{1}\left({\varvec{x}},{\Theta }_{1}\right), {f}_{2}\left({\varvec{x}},{\Theta }_{2}\right),\dots , {f}_{T}\left({\varvec{x}}, {\Theta }_{\mathrm{T}}\right)\}$, where now each tree has an additional parameter ${\Theta }_{t}$, the RF could be formally written as

2 $${f}_{RF}\left(x\right)=\frac{1}{T}{\sum_{t=1}^{T}}{\mathrm{\alpha }}_{t}{f}_{t}\left({\varvec{x}},{\it{\Theta}}_{{\it{t}}}\right)$$

where ${\alpha }_{t}$ is an associated weight. For the regression case, this weight is generally chosen as one.

## Methods

### Signal acquisition

**Figure 2:**
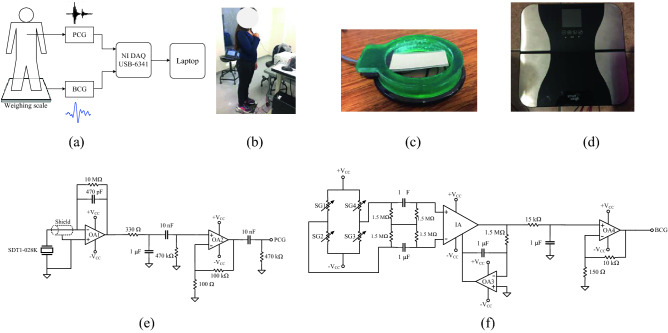
(**a**) Setup for detecting the PCG and the BCG simultaneously. (**b**) One of the volunteers using the measurement setup. (**c**) SDT1-028 K mounted on a custom-built case used for detecting the PCG. (**d**) Electronic weighing scale used for detecting the BCG. (**e**) Circuit used to detect the PCG, and (**f**) circuit used to detect the BCG. Figures (**a**, **e**, **f**) were drawn in Microsoft Visio Professional 2019^24^ by Rafael Gonzalez-Landaeta.

In this work, PCG and BCG were detected simultaneously (Fig. 2a,b). The PCG was detected at the pulmonary point located in the second intercostal space to the left of the sternum. The reason was that in preliminary tests, the PCG was obtained with greater amplitude at this point. The BCG was obtained by detecting cranial–caudal forces using an electronic weighing scale^23^.

To detect PCG, the approach proposed by Vazquez et al. was used^25^. A shielded piezofilm was used as a microphone (Fig. 2c) to obtain a high signal-to-noise ratio (SNR) PCG. For this, the SDT1-028K (TE Connectivity) was used and placed in a custom-built case to simplify the positioning of the sensor in the auscultation point. The conditioning circuit of the piezofilm consisted of a charge amplifier followed by a first-order passive bandpass filter and a non-inverting amplifier (Fig. 2e). The sensitivity of the entire system was 2.12 V/pC in a range of frequencies between 34 and 482 Hz. OA1 and OA2 were implemented using LT1793 (Linear Technology).

The BCG was detected using a weighing scale from Smart Weigh (Fig. 2d). The strain gauges (SG) mounted on the load cells of the scale formed a full Wheatstone bridge whose output was connected to the circuit shown in Fig. 2f. The total gain of the circuit was adjusted to 61 × 10^3^, with a bandwidth limited to between 0.5 and 10 Hz. The instrumentation amplifier (IA) used was the INA114 (Texas Instruments), and OA3 and OA4 were implemented using the TL082 (Texas Instruments). All the circuits were powered at ± 10 V with a Power Supply E3631A (Keysight). The PCG and BCG signals were registered using the data acquisition system USB-6341 (National Instrument) connected to a laptop and configured with a sampling frequency of 1 kHz.

### Signal processing

PCG is an acoustic signal that may be corrupted by noise from different sources, such as other sounds coming from the patient, skin contact with the stethoscope, and ambient noise^26–28^. Therefore, it was necessary to filter the PCG signal. For this study, it was decided to preserve components in the band above 34 Hz, since much of the low-frequency noise has main frequency components from 0 to 25 Hz^29^. In addition, because this study will not attempt to classify or detect sounds that indicate any abnormality, it was decided to eliminate high-frequency noises, unlike other studies in which pathologies are detected^30^. Therefore, the upper bound of the passband was established at 50 Hz, which facilitated the detection of sounds S1 and S2. Thus, the signal was filtered using a bandpass FIR filter designed with a Blackman window and cutoff frequencies of 34 Hz and 50 Hz. This also avoids other relatively high-frequency interferences, such as those produced by the AC line. In the case of BCG, this signal was filtered using a bandpass filter from 1 to 10 Hz, since it was observed that most of the signal power was in that band, which agrees with other studies^31^.

### Measurement of the features

The features considered in this study were the time intervals between S1-J, J-S2, S1-I, I-S2, K-S1, K-S2, the slope of the I-J segment, and the slope of the J-K segment. For the extraction of the features of interest, an algorithm similar to^32^ was used. Initially, all signals were normalized. Next, for the signals of each subject, the detection of the I, J, and K peaks was performed by comparing neighboring points to determine the local maxima and minima in the signal. Subsequently, using point J, which was previously detected, we proceeded to search for S1 in the PCG, looking for a local maximum before the temporary location of point J. Accordingly, point S2 was sought in the temporary neighborhood of point K. Finally, the peaks of waves I, J, and K of the next cycle were searched for at a distance of 4 ms from the previous J wave peak. This process was repeated until the end of the signal was reached. Then the time intervals between S1-J, J-S2, S1-I, I-S2, K-S1, K-S2, the slope of the I-J segment, and the slope of the J-K segment were calculated for each cardiac cycle. Finally, for each subject, a global estimate of each feature was obtained using the median of the measurements made for each cardiac cycle. The median instead of the sample mean was used since this is a more robust estimator of the mean of the real distribution, especially when measures are noisy and more prone to outliers^27^.

### Data analysis

#### Behavior of features regarding pressure changes induced by activities

With the measurements made on each individual, various statistical analyses were performed. The behavior of the features in response to changes in SBP was statistically analyzed using the empirical distributions of the features when the subjects performed different activities. Sample statistics of the mean and variance were calculated, as well as approximations to a probability distribution using kernel density estimation^28^. Moreover, to test the normality of the data, the Shapiro–Wilk test was used^33^.

#### Relationships of the features by distinctions in time and pressure

We further explored the relationships between these features. Based on the states of rest, activity, and post-activity, variations in pressure and the timing of characteristics were evaluated for the three states. The linearity of the relationship between the characteristics and the pressure was analyzed using the least squares regression technique with a Huber regularizer^27^, so the estimation was less influenced by outliers. To quantify the degree of association between the quantities involved, the Pearson correlation coefficient was calculated, which shows how well the data fit a linear relationship, and Spearman's correlation coefficient, which demonstrates how well the data agree with the monotone classification with relevant outliers^34^. The p-value was calculated based on the probability of the null hypothesis: that the current result would have been found if the correlation coefficients were zero. Therefore, it is assumed that if this probability is less than 5% (p-val < 0.05), the correlation coefficient will be statistically significant.

### BP estimation using machine learning algorithms

Once the features and their relationship with the SBP were analyzed, several machine learning algorithms were compared to estimate the SBP from the characteristics analyzed. It was determined which characteristics were the most predominant in the estimation process.

#### Dataset

A data set, ${D}_{1}={\{\left({x}_{i},{y}_{i}\right)\}}_{i=0}^{{N}_{1}}$, with ${N}_{1}=\mathrm{1,067}$, was used to train the machine learning algorithms, which consisted of records ${x}_{i}\in {\mathbb{R}}^{10}$ with features of the PCG and BCG signals. In addition, ${y}_{i}\in {\mathbb{R}}$ contains systolic pressure. To obtain the features, the measured signals of all subjects were segmented into cardiac cycles. A record ${x}_{i}$ then consisted of the following features obtained in one cycle: the intervals S1-J, J-S2, S1-I, I-S2, K-S1, K-S2, I-J, J-K, the slope from points I to J, and the slope from points J to K. Once the features were obtained, the corresponding systolic pressure was recorded in ${y}_{i}$. Notably, not all segmented cycles were used; those cycles where it was not possible to obtain all the features due to distortions or noise were eliminated. In addition, those records with S1-J intervals that were far from the mean by more than two standard deviations were discarded. Additionally, a second data set was contemplated, ${D}_{2}={\{\left({x}_{i},{y}_{i}\right)\}}_{i=0}^{{N}_{2}}$, with the same characteristics of ${D}_{1}$, except those records with measures of S1-J, S1-K, and I-J with a deviation of the mean by more than two standard deviations. This reduced the number of records to *N*_2_ = 235. The percentage of acceptance of cycles varied from person to person and with the activity; on average the percentage of acceptance for *D*_1_ was 78% and for *D*_2_ it was 41%.

#### Estimation of SBP

To estimate SBP, the following algorithms were evaluated: simple linear regression (LR); support vector machine (SVM), using a radial basis function kernel and parameters *c* = 45, gamma = 0.0001, epsilon = 0.01 for ${D}_{1}$ and *c* = 55, gamma = 0.0001, and epsilon = 0.01 for ${D}_{2}$; kernel ridge (KR), with the parameters alpha = 0.005, gamma = 0.05, and a Laplacian kernel function for ${D}_{1}$ and ${D}_{2}$; and RF, with 139 trees for ${D}_{1}$ and 210 trees for ${D}_{2}$. The algorithms were used in regression mode instead of classifier mode because the quantity to be estimated, the BP, is a non-categorical continuous variable. The parameters were optimized using the search grid method with fivefold cross validation on the training test, and all the algorithms were implemented with the sklearn library^35^*.* The algorithms were trained using data from 17 subjects, and data from four subjects were used for testing. The subjects in each set were chosen by a random permutation. It was decided not to use Deep Learning (DL) algorithms for two reasons: the tendency in DL is to obtain features automatically, using, for example, convolutional networks, which in some contexts such as medicine could make their interpretation difficult^36,37^, and second, these types of architectures generalize better when they are trained with a large amount of data^38^*.* Moreover, the selected algorithms have a low computational cost, which could facilitate their implementation in an embedded system, as they do not require a high-end processor for their programming.

To evaluate the regressors, the following metrics were used: the Explained Variance Score (EVS), which calculates an estimate of the explained variance by the algorithm used, and is given by

3 $$EVS=1-\frac{Var\left(y-\widehat{y}\right)}{Var\left(y\right)}$$

where $\widehat{y}$ is the value estimated by the regressor and $Var(.)$ is the variance of the argument. The coefficient of determination (*R*^2^) estimates the proportion of the variation in the dependent variable that is predictable from the independent variable, and is given by

4 $${R}^{2}=\frac{\sum_{i}{({y}_{i}-\widehat{{y}_{i}})}^{2}}{\sum_{i}{({y}_{i}-\overline{y })}^{2}}$$

where the variable *i* runs through all observations and $\overline{y }$ is the mean of the observations. The mean absolute error (MAE) and mean square error (MSE) were also used to evaluate the regressors.

### Measurement protocol

**Figure 3:**
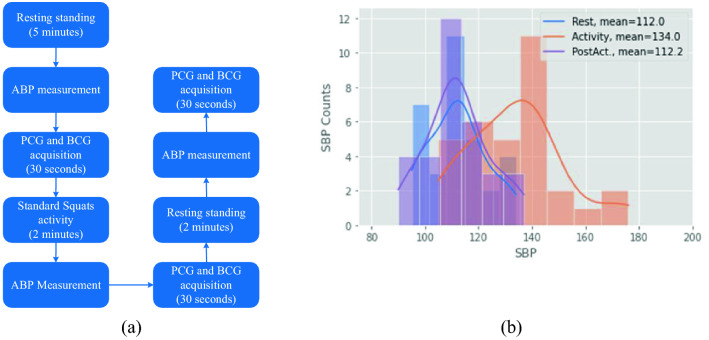
(**a**) Description of the measurement protocol. (**b**) Distribution of pressure measures related to each activity.

The PCG and the BCG of 21 healthy subjects (11 females and 10 males) were measured (mean ± SD): age = (23 ± 4) years, weight = (65 ± 12) kg, and height (169 ± 12) cm. Subjects with hypertension or other cardiac diseases were not considered in this study. The corresponding approval (CIEB-2019-1-106) from the institutional ethical committee of the Autonomous University of Ciudad Juarez was obtained, and a written informed consent was signed by the volunteers. All experiments were performed according to relevant guidelines and regulations. Figure 3a describes the measurement protocol for each subject. Two resting periods were considered: the first one of 5 min before the physical activity, and the second one of 2 min after the physical activity. The ABP of the volunteers and the biosignals were measured after each rest period. To induce variations in ABP, the volunteers performed standard squats for 2 min, after which the ABP and biosignals were measured. The ABP was measured with an automatic blood pressure monitor HEM-7200 (Omron), with an accuracy of ± 3 mmHg. The measurement protocol was repeated twice for each volunteer. Figure 3b reveals the distribution of the data collected with the measurement protocol. Data were summarized graphically using histograms of the samples and grouped by the subjects’ states during the measurement: rest, activity, and post-activity. In addition, approximations to a probability distribution using the kernel density estimation method^28^ are illustrated as continuous curves. As expected, the mean pressure during activity was higher than at rest and post-activity; in addition, the mean pressures at rest and post-activity were very similar.

## Results and discussion

**Figure 4:**
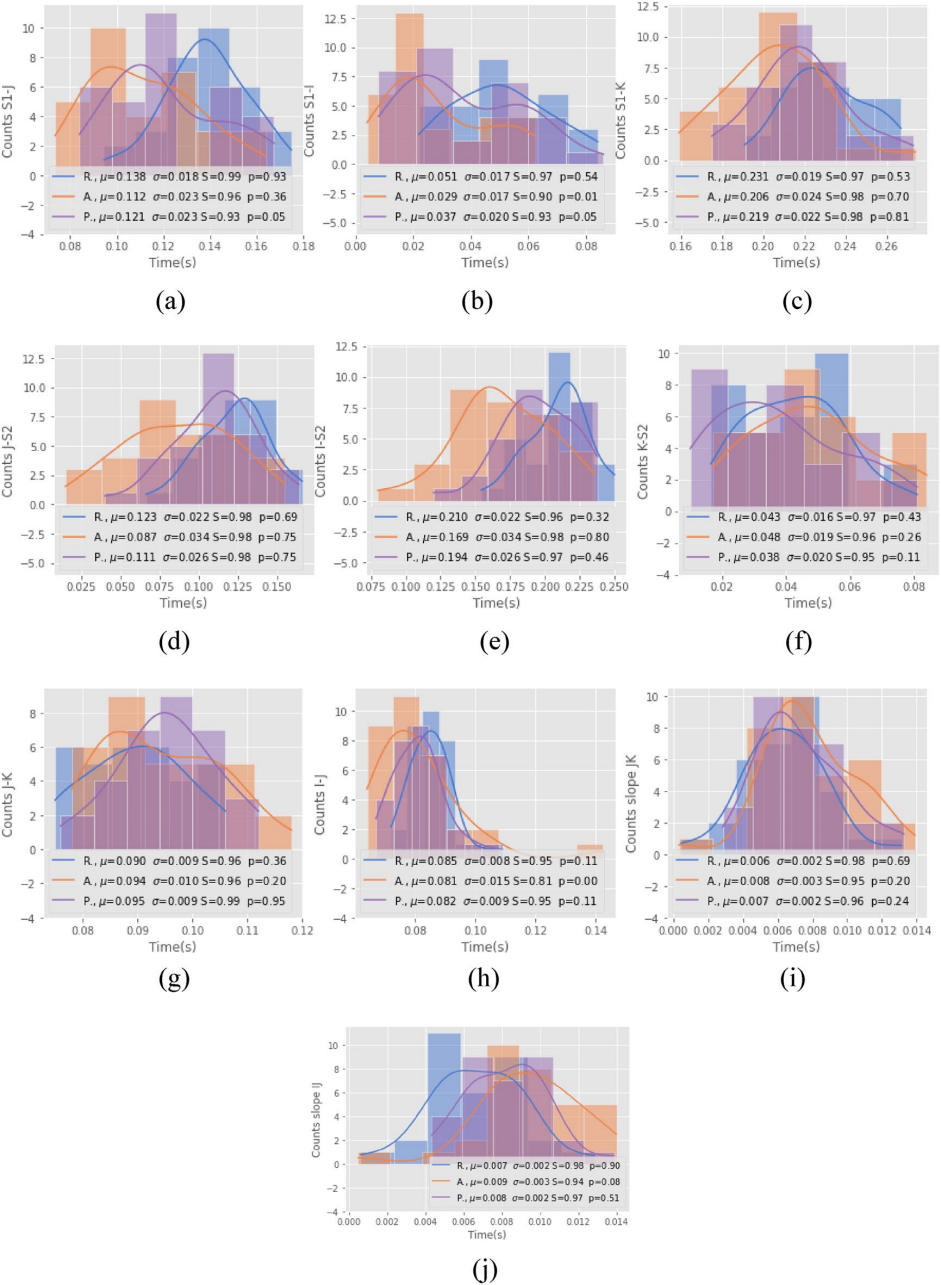
Histograms of the features according to the subject state: R = Rest, A = Activity, P = Post-activity, *μ* = Mean, *σ* = Std. dev., S = Shapiro test for normality, and p = p-value of the Shapiro test. Sample: 21 volunteers: (**a**) S1-J, (**b**) S1-I, (**c**) S1-K, (**d**) J-S2, (**e**) I-S2, (**f**) K-S2, (**g**) J-K, (**h**) I-J, (**i**) slope J-K, and (**j**) slope I-J.

Figure 4 illustrates the histograms for each of the features obtained and as an estimate of its probability distribution (continuous line). For each characteristic, three histograms were obtained depending on the person's state, that is, at rest, physical activity, and post-activity. The first row of Fig. 4 shows that, in the intervals S1-J, S1-I, and S1-K, the means of their empirical distributions followed the same pattern; as physical activity increases, the mean of the intervals decreases. In addition, in the measurements of the intervals J-S2 and I-S2 (second row of Fig. 4), despite the distributions being noisier, their means followed the same pattern, except for the interval K-S2, where the mean of the activity data is greater than that of the post-activity, which may be due to the dispersion in the data in both histograms, which revealed great variance with respect to their mean. In addition, it can be seen that the means of all the distributions are very close. Regarding the I-J and J-K intervals and the JK slope, the histograms were more defined, with less variance. In the I-J slope, their distributions presented a greater flattening at their peak. All the data had a normality statistic above 0.8, according to the Shapiro–Wilk test. However, for the case of the I-J interval, the physical activity data did not reach statistical significance.

**Figure 5:**
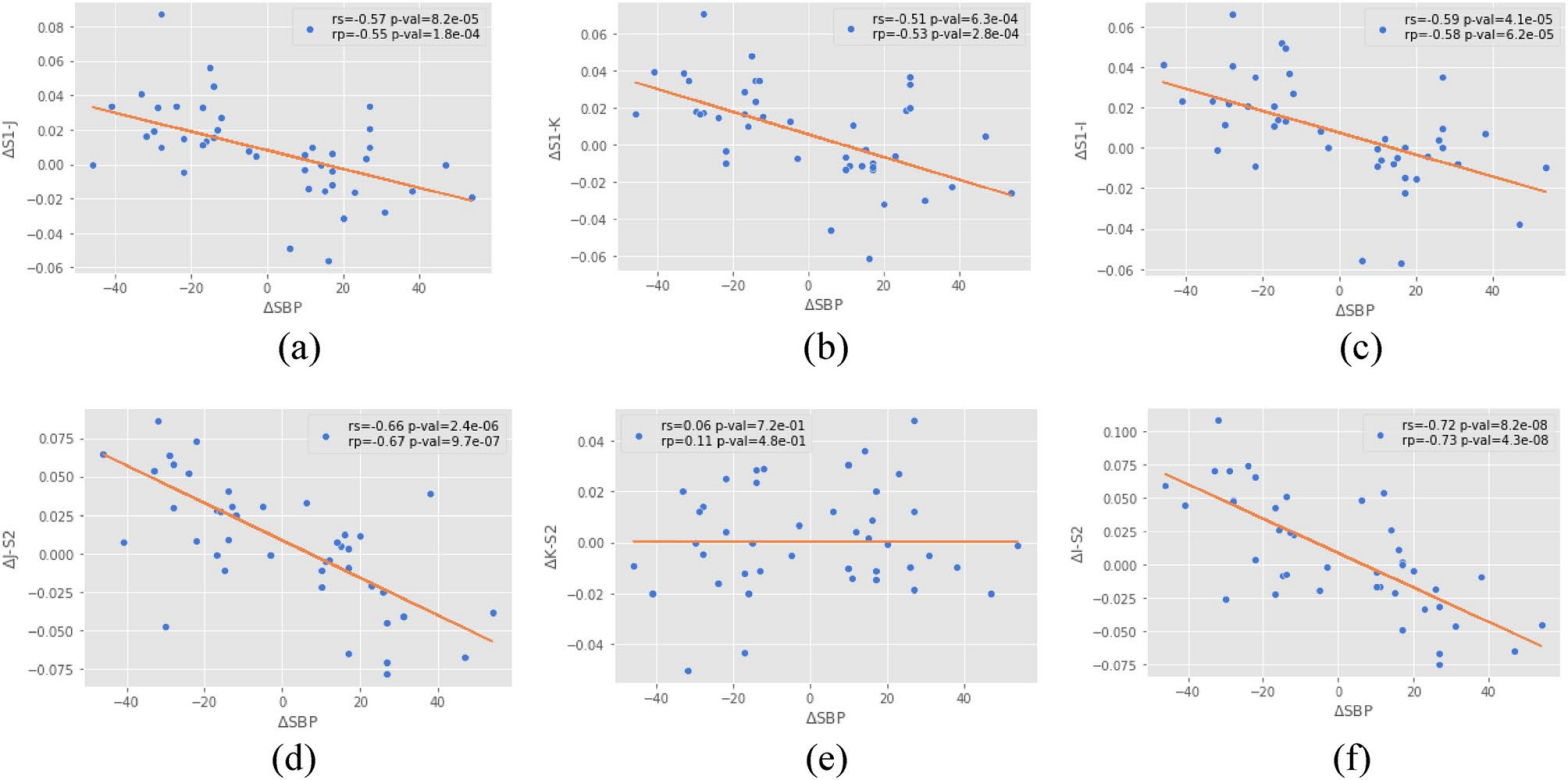
Scatter plots of time intervals against SBP changes in the features for different states. rs = Spearman correlation coefficient; rp = Pearson correlation coefficient.

Figure 5 illustrates scatter plots of time intervals against SBP changes for all features and for different states. For each feature, correlation coefficients and the regression curve were calculated. For this, the Spearman and Pearson correlations were estimated, as well as their p-values. The results of this study showed that the intervals S1-J, S1-K, S1-I, I-S2, and J-S2 were negatively correlated with changes in SBP (p-val < 0.01). However, for the difference S2-K, a slightly positive correlation trend and a greater dispersion in the data existed. With a p-val > 0.01, its statistical significance was ruled out.

Once the proposed characteristics were analyzed, they were used as explanatory variables for training the regression algorithms. Table 1 portrays the evaluation of the regression algorithms on the test set. For the case of using all measures, *D*_1_, SVM was the least adapted; even though various kernels were tested, the best kernel *rbf* could not model the non-linearity of the characteristics. The regressor based on Random Forest achieved the best adaptation with the least outliers; this was observed in the lowest MSE for all methods. Regarding the use of data, *D*_2_, constrained by the standard deviation, however, the other regressors also obtained good performance, especially the linear regression model.

**Table 1:** Evaluation of the regression algorithms with the metrics.

	*D*_1_ dataset				*D*_2_ dataset			
	EVS	R^2^	MAE	MSE	EVS	R^2^	MAE	MSE
LR	0.302	0.040	8.072	125.89	0.719	0.332	5.646	46.06
KR	0.973	0.039	13.21	267.54	− 0.190	− 0.021	7.362	70.53
SVM	0.319	0.192	9.391	156.55	0.598	0.216	5.799	54.14
RF	0.318	0.213	7.090	103.26	0.641	0.486	4.402	35.47

**Figure 6:**
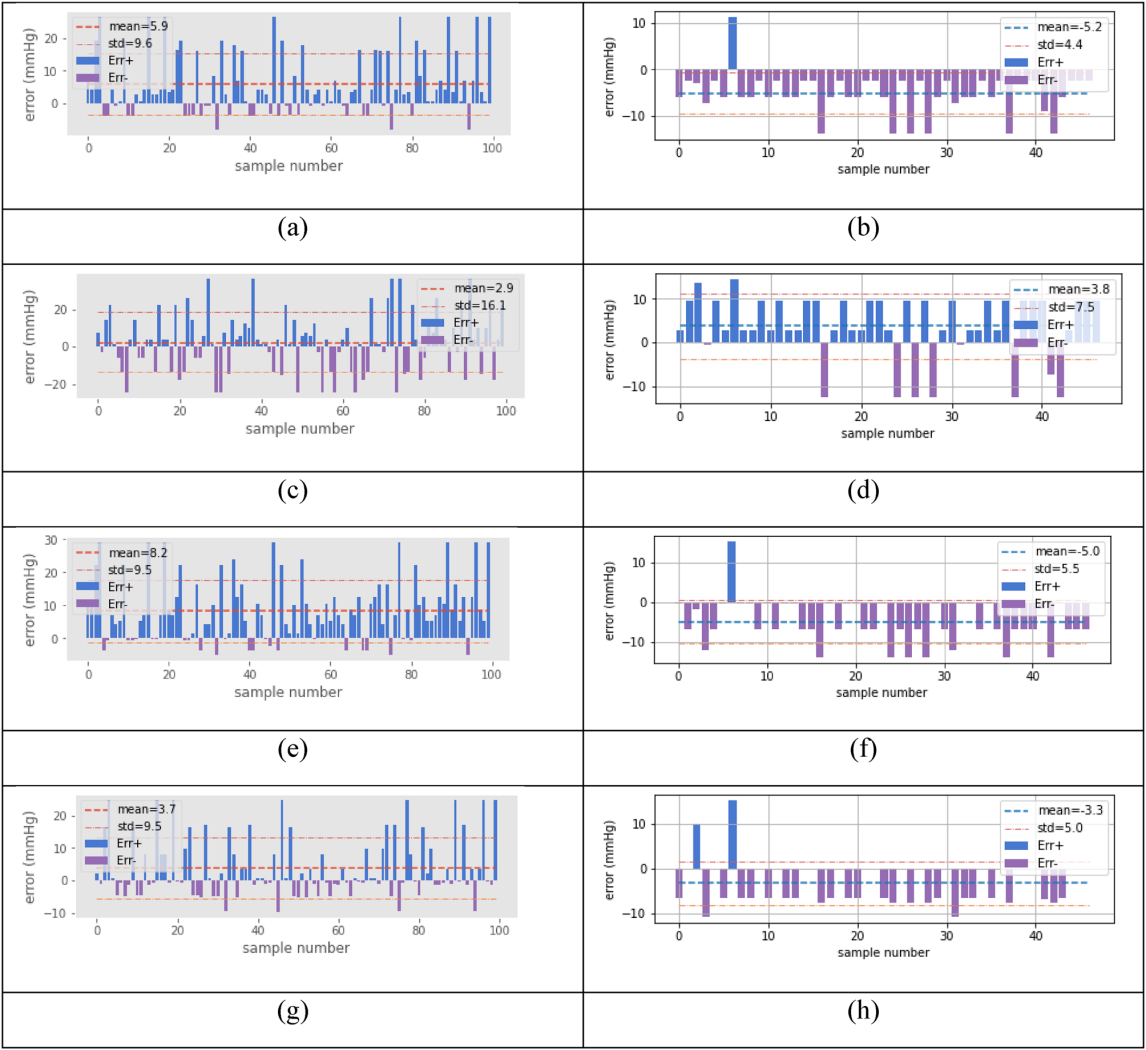
Error in units of pressure, left column, reveals the performance using all measures, and the right column uses restricted data. First line (**a**), (**b**) linear regression, second line (**c**), (**d**) ridge regression, third line (**e**), (**f**) SVM, and fourth line (**g**), (**h**) Random Forest.

**Figure 7:**
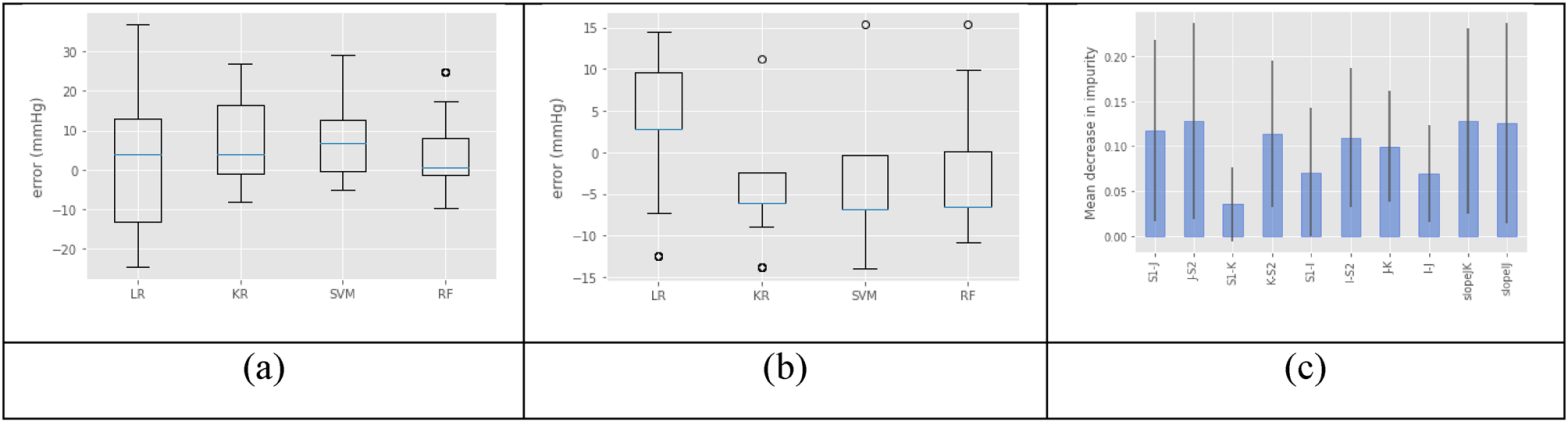
Boxplots: (**a**) Four algorithms evaluated in *D*_1_, (**b**) Four algorithms evaluated in *D*_2_, and (**c**) Feature importance based on MDI.

Figure 6 shows error graphs for the algorithms. For the case of using the dataset ${D}_{1}$, the first column of Fig. 6, the best mean (2.9) is obtained by the KR. However, many measurements exceed the error by more than 20 units, which is reflected in a high MSE. The second-best method is the RF, which obtains a mean error of 3.7, where few measurements have more than 20 units of error compared to the other methods. The same occurs with LR, but to a lesser extent. As for SVR, it has the poorest performance of the exposed methods. Notably, for the clarity of graphs, only the first 100 samples are shown. Nonetheless, the mean and standard deviations were calculated using the entire ${D}_{1}$ dataset. Figure 7a demonstrates a boxplot of the statistics of each algorithm.

Regarding dataset ${D}_{2}$, the RF obtains the smallest mean error of 3.3, followed by KR, though the latter presents more values greater than 20, so its MSE is large. Furthermore, in the RF method, several predicted values are zero, which does not occur in the other methods. Figure 7b depicts boxplots of the performance of the algorithms on the ${D}_{2}$ dataset.

Finally, using the best classifier (RF in *D*_2_), the importance of the features was analyzed. In Fig. 7c, the bar height shows the importance in the Random Forest, while the lines show the variability between trees represented by the error bars. The importance is based on the mean decrease in impurity (MDI). The most important features are the intervals J-S2, followed by the slope J-K and slope IJ. Interestingly, point J is involved in two of the three features. Conversely, the feature with the least weight is the S1-K interval, which could be due to the distance between the two points.

## Conclusion

In this work, a new cuffless method for estimating systolic blood pressure was proposed. It is not based on pulse transit time or pulse arrival time estimation, but relies on analyzing the correlation between the S1 and S2 of the phonocardiogram and the I, J, and K waves of the ballistocardiogram, which can be detected noninvasively in a simple way. For this, various intervals between these two signals were used as features to train the machine-learning algorithms, including the IJ and JK slopes of the ballistocardiogram. The means of the empirical distributions of the intervals S1-J, S1-I, and S1-K decreased as the systolic blood pressure increased. The same behavior was observed for the J-S2, I-S2, and K-S2 intervals, although with a noisy probability distribution. Except for the interval K-S2, these features were negatively correlated with the systolic pressure, with a p-val < 0.01. Among all the regression algorithms employed, Random Forest exhibited a coefficient of determination of 0.48, and the mean error (mean difference) was 3.3 mmHg with a standard deviation of ± 5 mmHg in the estimation of systolic blood pressure. According to the mean decrease impurity, the best features to estimate systolic blood pressure were the S1-K interval and the JK slope of the BCG, which can also be used as indicators of changes in systolic blood pressure. From the results presented here, correlating the PCG and the BCG signals could be used to estimate changes in systolic blood pressure, and this could be a starting point to implement wearable systems that do not require pulse detection in peripheral arteries. Our vision is to develop a compact device capable of estimating blood pressure non-invasively by simultaneously detecting both PCG and BCG in a single area of the body (chest). This not only simplifies the blood pressure measurement procedure but may be useful for subjects with partial or total amputations of some or all of their limbs, and who cannot use current cuffless methods.

## Data Availability

The datasets generated during the current study are available in the MAYAS project repository, https://github.com/MAYASproject/data.
